# Culture-independent molecular analysis of bacterial diversity in uranium-ore/-mine waste-contaminated and non-contaminated sites from uranium mines

**DOI:** 10.1007/s13205-011-0034-4

**Published:** 2011-11-03

**Authors:** Paltu Kumar Dhal, Ekramul Islam, Sufia K. Kazy, Pinaki Sar

**Affiliations:** 1 Department of Biotechnology, Indian Institute of Technology, Kharagpur, 721 302 India; 2 Department of Biotechnology, National Institute of Technology, Durgapur, 713 209 India

**Keywords:** Uranium mines, 16S rRNA gene, Bacterial community, ARDRA

## Abstract

Soil, water and sediment samples collected from in and around Jaduguda, Bagjata and Turamdih mines were analyzed for physicochemical parameters and cultured, and yet to be cultured microbial diversity. Culturable fraction of microbial community measured as Colony Forming Unit (CFU) on R2A medium revealed microbes between 10^4^ and 10^9^ CFU/g sample. Community DNA was extracted from all the samples; 16S rRNA gene amplified, cloned and subject to Amplified Ribosomal DNA Restriction Analysis. Clones representing each OTU were selected and sequenced. Sequence analyses revealed that non-contaminated samples were mostly represented by *Acidobacteria*, *Bacteroidetes*, *Firmicutes* and *Proteobacteria* (*β*-*, γ*-*,* and/or *δ*-subdivisions) along with less frequent phyla *Nitrospira*, *Deferribacteres*, *Chloroflexi*. In contrast, samples obtained from highly contaminated samples showed distinct abundance of *β*-*,γ*- and *α*-*Proteobacteria* along with *Acidobacteria,Bacteroidetes* and members of *Firmicutes, Chloroflexi*, *Candidate division, Planctomycete*, *Cyanobacteria* and *Actinobacteria* as minor groups. Our data represented the baseline information on bacterial community composition within non-contaminated samples which could potentially be useful for assessing the impact of metal and radionuclides contamination due to uranium mine activities.

## Introduction

Dispersion and migration of uranium (U) and other toxic metals and radionuclides from uranium mines due to mining operations and waste piling is a serious environmental concern to all uranium producing states (Foster et al. 2008; Islam et al. 2011). Once released into the environment, fate and toxicity of these metallic contaminants are strongly regulated by abiotic and biotic components such as minerals and bacteria (Lloyd and Renshaw 2005). On the other hand, the toxic and bioavailable forms of these metals and radionuclides often affect adversely the diversity and function of autochthonous microorganisms of neighboring habitats that bequeath ecological sustains by maintaining biogeochemical cycles (Torsvik et al. 2002; Islam et al. 2011). Diversity and distribution of microbes is often site-specific, influenced by composition of geochemical matrix of microhabitats. Perturbation to this due to environmental contamination could cause change in inhabitant microbial community structure, diversity and function (Herrera et al. 2007; Desai et al. 2009). In order to gauge the impact of environmental contamination, microbial community composition and diversity are increasingly being considered as highly sensitive ecological parameters (Wang et al. 2007; Desai et al. 2009; Islam and Sar 2011b). Therefore, studies on diversity and composition of indigenous microbial communities within sites having high risk of contamination may serve as baseline information to assess the subsequent impact of contamination.

In the recent years, advances in culture-based and -independent molecular approaches have elucidated microbial diversity and function in sites contaminated with radioactive wastes or wastes generated from uranium, copper and zinc mines. These studies have revealed the presence of diverse (phylogenetically and metabolically) populations of viable and active microorganisms organized in complex communities (Radeva and Selenska-Pobell 2005; Akob et al. 2007; Rastogi et al. 2010). Particularly, the abundance of *Acidithiobacillus*, *Pseudomonas, Acinetobacter*, *Nitrosomonas* was observed in various U mine waste sites in Germany (Radeva and Selenska-Pobell 2005), while high abundance of *Proteobacteria* (*Sphingomonas*, *Acidovorax*, *Acinetobacter* and *Ralstonia*) was reported from U-contaminated radioactive waste (Akob et al. 2007). Recently, Rastogi et al. (2010) has reported abundance of *Proteobacteria*, *Acidobacteria* and *Bacteriodetes* in a U mine impacted site in USA. Studies conducted in our laboratory have revealed microbial communities within two U mines (Jaduguda and Banduhurang) along with potential of inhabitant bacteria in U and other metal resistance and sequestration and impact of U ore contamination on soil microbial diversity (Sar et al. 2007; Choudhary and Sar 2011; Islam et al. 2011; Islam and Sar 2011a, b). With vast genetic and metabolic diversity, these microorganisms were found to interact with metals and radionuclides directly or indirectly by redox transfer, biosorption, bioaccumulation or bioprecipitation affecting their environmental mobility and toxicity (Suzuki and Banfield 2000; Tabak et al. 2005; Nedelkova et al. 2007). Considering the significance of geomicrobiology of contaminated sites, it is therefore imperative to decipher phylogenetic diversity of indigenous microbial populations in sites having high risk of contamination or already contaminated to illuminate community resilience, their potential role in affecting metal biogeochemistry and in designing appropriate bioremediation strategies (Tabak et al. 2005; Akob et al. 2007).

Uranium mines at Jaduguda, Bagjata, and Turamdih are all located in highly mineral-rich areas of East Singhbhum district, Jharkhand, India. Jaduguda mine is the oldest U mine in India operating since 1968, while the other two mines are commissioned relatively recently (2002–2003) (Gupta and Sarangi 2005). Present work was undertaken to ascertain diversity and structure of bacterial communities within sites in and around these U mines as a means to obtain the baseline data on microbial diversity. Sites contaminated with U-ores/-mine wastes as well as sites located away from such contaminants (ores and wastes; especially, agriculture fields, streams, etc. located outside the mine areas) were considered. Samples collected from all these sites were analyzed for their physicochemical properties (pH, conductivity, total organic carbon (TOC), total nitrogen (TN), total phosphorus (TP) and heavy metal content). Culturable bacterial counts were recorded and finally the compositions of microbial communities were determined at molecular level with the determination of diversity indices and identification of dominant ribotypes.

## Materials and methods

### Collection of samples

Soil, water and sediment samples were collected from various locations of proposed and existing uranium mines of at Jaduguda (N 22°39′, E 86°20′), Bagjata (N 22°28′, E 86°29′), and Turamdih (N 22°43′, E 86°11′). Samples B209, B210, B211, B214, B218 and T112, T219, T221, T222 were collected from agriculture field; pond and small streams located outside the boundary of Bagjata and Turamdih mines, respectively, and are designated as non-contaminated samples. Samples CW1, CW2 and CW3 were obtained from sites within the mines and contaminated with mine wastes and ores and are designated as contaminated samples. All samples were collected aseptically and stored immediately in ice till further analysis.

### Geomicrobiological analysis

Physiochemical parameters including pH, conductivity/salinity of the samples were measured by an Orion star™ series meter (Thermo Electron Corporation) following the procedure as described elsewhere (Islam and Sar 2011a). Heavy metals and actinide elements were estimated by ICP-MS (Varian) and/or AAS (Perkin-Elmer). Culturable microbial populations were enumerated by CFU counts using R2A medium. Measured quantities of samples were dispersed in sterile saline (0.9%), shake (1 h), diluted and plated in triplicate on R2A medium. The composition of R2A medium was (g/lit): yeast extract, 0.5; peptone, 0.5; casamino acid, 0.5; dextrose, 0.5; soluble starch, 0.5; sodium pyruvate, 0.3; dipotassium phosphate, 0.3; magnesium sulphate, 0.05; (Fredrickson et al. 2004). Plates were incubated at 30 °C in dark and colonies were counted after 1 week.

### Extraction of community metagenomes, PCR amplification of 16S rRNA genes, cloning and clone library analysis

Community metagenome from each sample was extracted using Power soil™ DNA kit (MO BIO laboratories, Inc.). 16S rRNA genes from each extracted metagenomes were PCR amplified using universal and bacteria-specific primers (27F and 1492R, respectively) (Islam et al. 2011). Amplified products were purified using gel-purification kit (Qiagen), cloned into pGEM-T easy vector (Promega) and transformed into *E.coli* JM109 following manufacturer’s instructions. For each sample, positive clones were selected for clone library construction. Detailed description of methodology was same as described previously (Islam and Sar 2011a).

### Amplified ribosomal DNA restriction analysis (ARDRA)

From each library 50–100 clones were used for reamplification of cloned 16S rRNA gene by colony PCR with vector-specific primers. Colony PCR products were purified and digested with restriction enzymes in separate reactions. Portion (10 μl) of the amplification products were digested at 37 °C for 3 h with the restriction endonuclease *Rsa*I and *Hae*III. Digestion products (10 μl) were run on 2.5% agarose gel for 3 h and band patterns were compared visually.

### Phylogenetic analysis 16S rRNA gene

All positive 16S rRNA gene clones were grouped according to their ARDRA pattern and each group was referred as an operational taxonomic unit (OTU). About first 600–700 nucleotides of representative clones from each dominant OTU were sequenced. All sequences were examined as potential chimeras using the CHIMERA_CHECK program at the Ribosomal Database Project II (RDP-II) with default settings. Both BLAST program of NCBI database and Hierarchy Browser from Ribosomal Database Project II (RDP-II) were used to find out nearly identical sequences for the 16S rRNA gene sequences determined. Phylogenetic trees were constructed using the Neighbor-joining method with Jukes-Cantor distance correction in MEGA software version 4 using selected sequences (>500 nucleotides and positive polarity) (Tamura et al. 2007).

### Statistical analysis

Based on ARDRA profiles, Shannon’s equitability and diversity indices were calculated for each sample (Shannon 1948).

### Nucleotide sequence accession numbers

Nucleotide sequences obtained in the present study were deposited in GenBank database under the accession numbers HM469536-HM469615 and HQ909283-HQ909337.

## Results and discussion

### Physicochemical and microbiological analyses

**Fig. 1:**
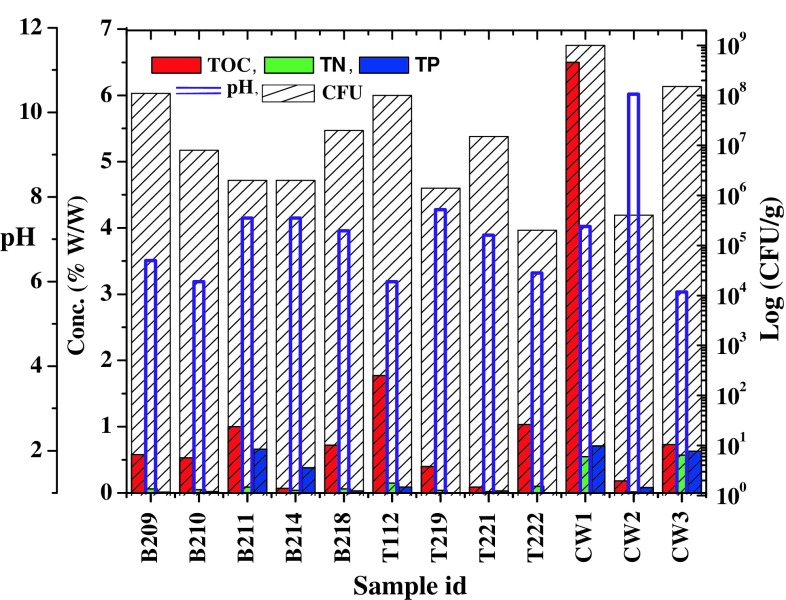
Geomicrobial properties of the samples

**Fig. 2:**
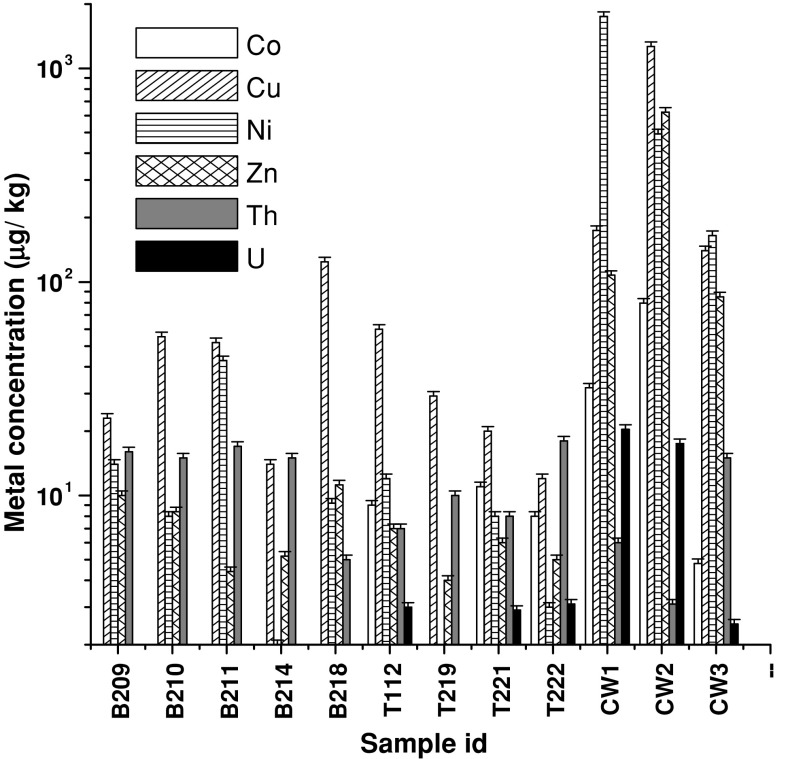
Analysis of heavy metals and radionuclides (U and Th) content within the samples

All samples were analyzed for their physicochemical and microbiological properties (Figs. 1, 2). As evident from the Fig. 1, samples were mostly having near neutral pH (except a few, e.g., B209, B210, T112, T222 with relatively lower pH and CW2 having an alkaline pH). Conductivity of the contaminated samples showed considerably higher values (955–4,340 μs/cm) than that of non-contaminated samples (10–244) (data not shown). It was observed that overall nutrient (TOC, TN and TP) content in contaminated samples, particularly in CW1 and CW3, was relatively higher than other contaminated and even non-contaminated samples. Microbiological counts as obtained by CFU counts were higher in few samples like B218, T221, CW1 and CW3. Heavy metal content as analyzed in non-contaminated samples indicated that for the elements tested the concentrations were within the limits for background concentrations of trace elements estimated in non-anthropogenic soils (Burt et al. 2003). Level of Th was found to be within a relatively narrow range of 3–17 mg/kg across all the samples tested. Concentrations of metals like Co, Ni and Zn were considerably higher in contaminated samples (over the non-contaminated counterparts) exceeding the values reported for non-anthropogenic soils or matches well with those reported for anthropogenically contaminated soils (Burt et al. 2003). Uranium, though present in several non-contaminated samples as well, was present at elevated level in two contaminated samples (CW1 and CW2) (Fig. 2). Noticeably, in all three non-contaminated samples simultaneous presence of three or more metals (including U) at higher concentrations was observed. Presence of U and other metals in non-contaminated samples could be explained considering the fact that this whole region is highly mineral-rich and natural weathering may easily enrich the soil and other parts with metals (Sarangi and Singh 2006).

### 16S rRNA gene clone library analysis and bacterial diversity indices

For analyzing bacterial diversity nearly 50 or 100 clones were used to construct individual clone library for each sample. Based on ARDRA profiles, statistical analysis was followed to determine the bacterial diversity within these samples. Interestingly, all three contaminated samples showed relatively higher Shannon diversity indices (*H*) but the equitability value (*E*_*H*_) varied considerably among the samples [B209 (2.88, 0.71), B210 (3.0, 0.93), B211 (2.77, 0.89), B214 (2.48, 0.63), B218 (2.99, 0.94), T112 (3.22, 0.95), T219 (3.52, 0.97), T221 (2.6, 0.70), T222 (2.9, 0.95), CW1 (3.65, 0.92), CW2 (3.51, 0.89) and CW3 (3.69, 0.92)]. Based on diversity result, we inferred that high nutrient content of the samples possibly make the heavy metal contamination as less stressful event as the inhabitant bacterial members might be nutritionally well supported to withstand metal toxicity. Nevertheless, near neutral pH may allow formation of relatively insoluble metal complexes for most cations thereby reducing their availability as well.

### Sequence analysis and affiliation of major ribotypes

**Fig. 3:**
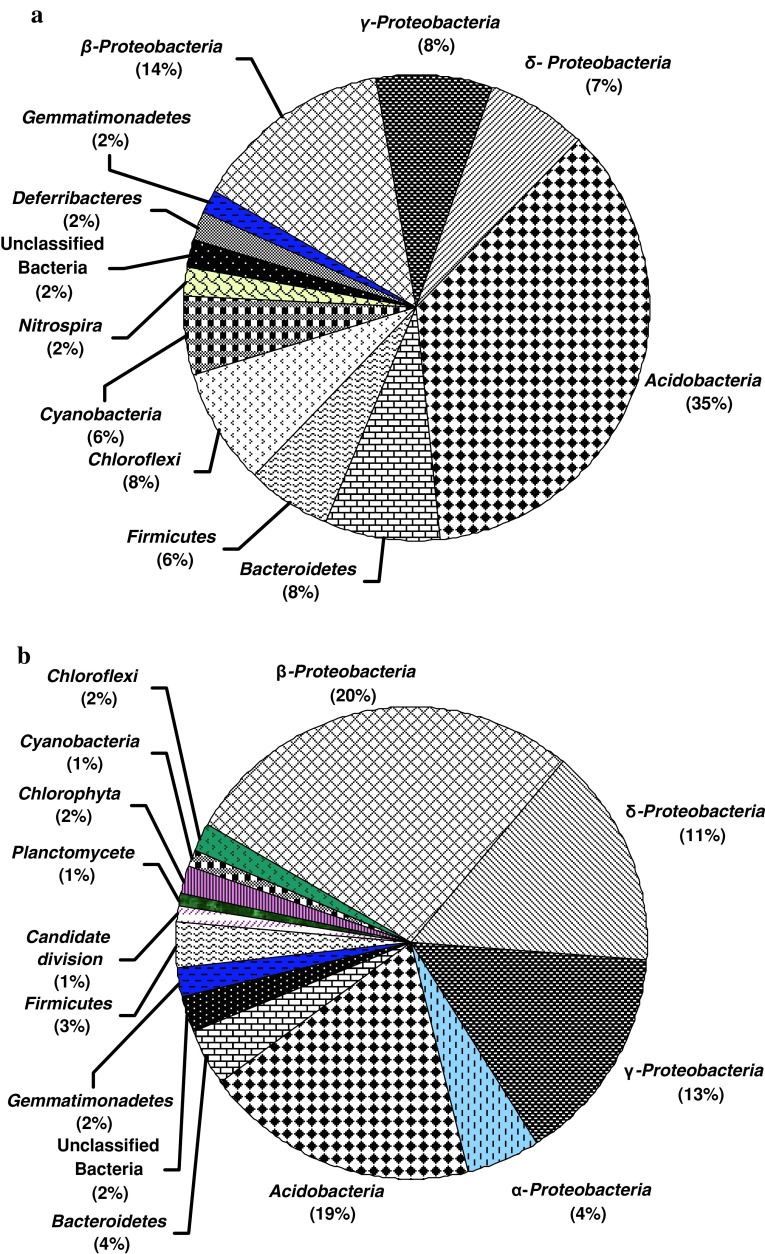
Distribution of different bacterial groups within (**a**) non-contaminated and (**b**) contaminated samples

**Fig. 4:**
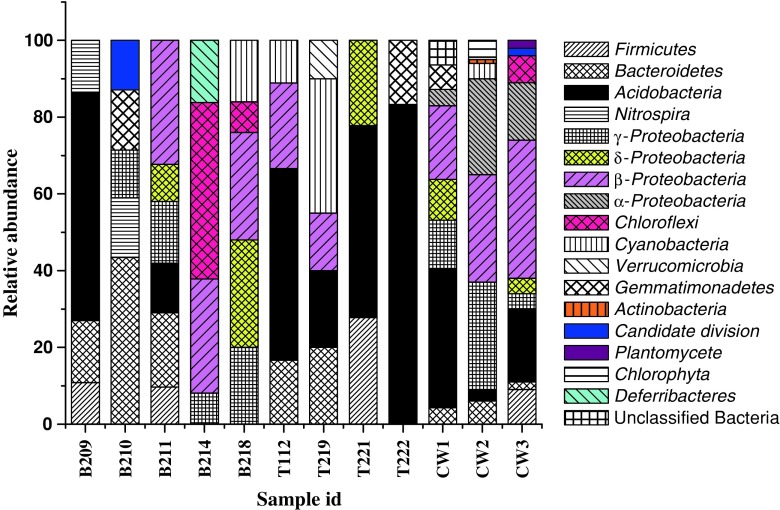
Relative abundance of bacterial phyla detected within the samples

Compositions of bacterial communities within the samples were ascertained by analyzing 16S rRNA gene sequences of major ribotypes from each library (Figs. 3a, b and 4). Our analysis revealed that samples from non-contaminated sites were mostly represented by *Acidobacteria*, *Bacteroidetes*, *Firmicutes* and *Proteobacteria* (*β*-*, γ*-*,* and/or *δ*-subdivisions) along with less frequent *Nitrospira*, *Deferribacteres*, *Chloroflexi* in one or few samples. Among the samples collected from Bagjata, bacterial communities in B209 (agriculture field) and B211 (river sediment) showed representatives of *Bacteroidetes*, *Acidobacteria* and *Firmicutes*. Presence of *β*-, *γ*- and *δ*-*Proteobacteria* in B211 was noticeable. Sample B210 (pond sediment) comprised *Bacteroidetes*, *Gemmmatimonadetes* and Candidate division along with *γ*-*Proteobacteria*. Although members of *β*- and *γ*-*Proteobacteria* were found in B 214 (garden soil) and B218 (river sediment), the absence of all three major phyla like *Acidobacteria*, *Bacteroidetes* and *Firmicutes* was notable. Additionally, phyla *Chloroflexi* and *Deferribacteres* were present in high percentage in B214, while *Cyanobacteria* and *Chloroflexi* were present in B218. All the samples from Turamdih showed the presence of *Acidobacteria* as a major group along with *Bacteroidetes*/*Firmicutes*/*Cyanobacteria* or *Gemmmatimonadetes* in one or more samples. Members of *Proteobacteria* were not so abundant in these samples. Particularly, *γ*-*Proteobacteria* was not found, although the presence of either *β*- or *δ*-*Proteobacteria* was detected in few samples.

Samples obtained from highly contaminated locations of Jaduguda mine showed distinct abundance of *β*-*Proteobacteria,* along with *γ*- and *α*-*Proteobacteria*, *Acidobacteria,Bacteroidetes* and *Firmicutes*. Members of *Actinobacteria, Chloroflexi, Planctomycetes*, *Cyanobacteria* and *Gemmmatimonadetes* were detected as relatively minor groups. Sample CW1 showed diverse assemblage of bacterial populations well represented by *Proteobacteria* (*β*- > *γ*- > *δ*- > *α*-subdivisions) > *Acidobacteria* > *Gemmmatimodadetes* > unclassified bacteria > *Bacteroidetes*. The observed community structure within this sample corroborates well with its relatively higher organic carbon, nitrogen and phosphorous content and CFU counts suggesting that in spite of higher metal contamination bacterial flora can flourish very well possibly by developing appropriate homeostatic mechanisms (Akob et al. 2008).

### Phylogenetic analysis of microbial groups

**Fig. 5:**
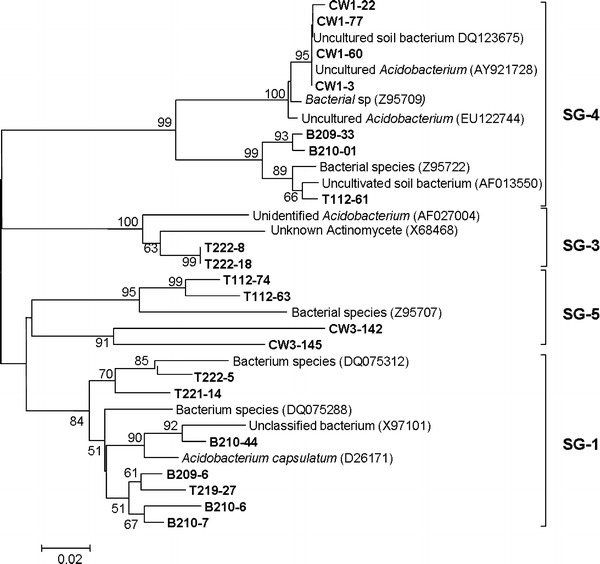
Phylogenetic dendrogram of the *Acidobacterium* division based on Neighbor-joining analysis. Subdivisions (see the text) are indicated in *brackets* at the right of the tree

**Fig. 6:**
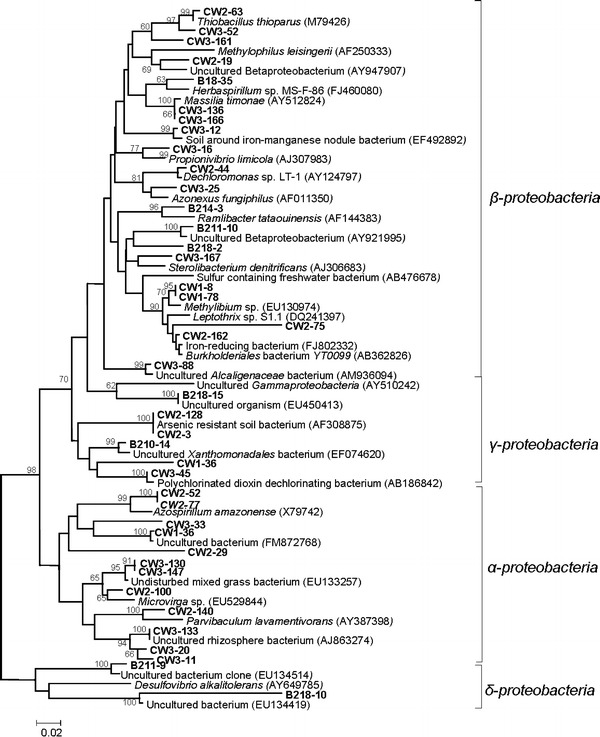
Neighbor-joining tree constructed based on proteobacterial 16S rRNA gene sequences detected in the present study along with similar sequences retrieved from NCBI and RDP databases. *Numbers* at nodes indicate percent bootstrap values above 80 supported by 1,000 replicates. *Bar* indicates Jukes-Cantor evolutionary distance

**Fig. 7:**
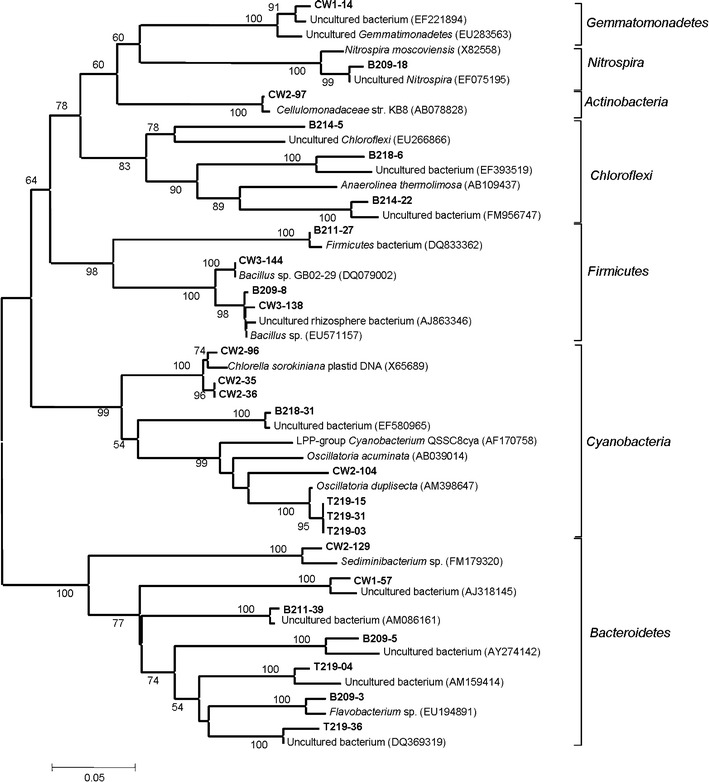
Neighbor-joining tree constructed based on 16S rRNA gene sequences of phyla *Bacteroidetes*, *Firmicutes*, *Actinobacteria*, *Gemmatimonadetes* and *Cyanobacteria* detected in the present study along with similar sequences retrieved from NCBI and RDP databases. *Numbers* at nodes indicate percent bootstrap values above 80 supported by 1,000 replicates. *Bar* indicates Jukes-Cantor evolutionary distance

Sequences representing ARDRA OTUs were analyzed to ascertain their phylogenetic lineage with similar taxa/groups from diverse ecological habitats and with environmentally relevant metabolic functions (Table 1; Figs. 5, 6, and 7).

**Table 1:** Details of 16S rRNA gene sequences retrieved in this study and used for phylogenetic analysis

Sl no.	Accession number	Clone id	Phylum	Present in
1	HM469555	T112-61C1	Acidobacterium	Fig. 5
2	HM469561	B209-3B2	Bacteroidetes	Fig. 7
3	HM469565	B209-18B5	Nitrospira	Fig. 7
4	HM469566	B209-3B6	Bacteroidetes	Fig. 7
5	HM469560	B209-8B1	Firmicutes	Fig. 7
6	HM469567	B209-33B8	Acidobacteria	Fig. 5
7	HM469568	B209-35B9	Acidobacteria	Fig. 5
8	HM469570	B210-6S4	Acidobacteria	Fig. 5
9	HM469571	B210-7S5	Acidobacteria	Fig. 5
10	HM469573	B210-14S1	γ-Proteobacteria	Fig. 6
11	HM469574	B210-44S6	Acidobacteria	Fig. 5
12	HM469575	B210-1S8	Acidobacteria	Fig. 5
13	HM469578	B211-9R5	δ-Proteobacteria	Fig. 6
14	HM469579	B211-39R2	Bacteroidetes	Fig. 7
15	HM469580	B211-27R7	Firmicutes	Fig. 7
16	HM469581	B211-10R1	β-Proteobacteria	Fig. 6
17	HM469584	B214-22N1	Chloroflexi	Fig. 7
18	HM469585	B214-3N2	β-Proteobacteria	Fig. 6
19	HM469586	B214-5N3	Chloroflexi	Fig. 7
20	HM469591	B218-31E1	Cyanobacteria	Fig. 7
21	HM469594	B218-6E4	Chloroflexi	Fig. 7
22	HM469597	B218-10	δ-Proteobacteria	Fig. 6
23	HM469592	B218-15E2	γ-Proteobacteria	Fig. 6
24	HM469593	B218-2E3	β-Proteobacteria	Fig. 6
25	HM469596	B218-35E7	β-Proteobacteria	Fig. 6
26	HM469598	T219-15D1	Cyanobacteria	Fig. 7
27	HM469600	T219-36D3	Bacteroidetes	Fig. 7
28	HM469601	T219-04D8	Bacteroidetes	Fig. 7
29	HM469602	T219-27D5	Acidobacteria	Fig. 5
30	HM469603	T219-31D4	Cyanobacteria	Fig. 7
31	HM469604	T219-03D7	Cyanobacteria	Fig. 7
32	HM469610	T221-14M5	Acidobacteria	Fig. 5
33	HM469612	T222-8G2	Acidobacteria	Fig. 5
34	HM469613	T222-5G3	Acidobacteria	Fig. 5
35	HM469614	T222-18G5	Acidobacteria	Fig. 5
36	HM469538	CW1-8A4	β-Proteobacteria	Fig. 6
37	HM469540	CW1-60A8	Acidobacteria	Fig. 5
38	HM469536	CW1-14A2	Gemmatimonadetes	Fig. 7
39	HM469543	CW1-3A11	Acidobacteria	Fig. 5
40	HM469550	CW1-77A19	Acidobacteria	Fig. 5
41	HM469541	CW1-36A9	α-Proteobacteria	Fig. 6
42	HM469545	CW1-22A13	Acidobacteria	Fig. 5
43	HM469548	CW1-56A17	γ-Proteobacteria	Fig. 6
44	HM469551	CW1-78A20	β-Proteobacteria	Fig. 6
45	HM469549	CW1-57A18	Bacteroidetes	Fig. 7
46	HQ909289	CW2-36	Cyanobacteria	Fig. 7
47	HQ909290	CW2-44	β-Proteobacteria	Fig. 6
48	HQ909293	CW2-77	α-Proteobacteria	Fig. 6
49	HQ909288	CW2-35	Cyanobacteria	Fig. 7
50	HQ909294	CW2-96	Cyanobacteria	Fig. 7
51	HQ909295	CW2-97	Actinobacteria	Fig. 7
52	HQ909296	CW2-128	γ-Proteobacteria	Fig. 6
53	HQ909324	CW3-166	β-Proteobacteria	Fig. 6
54	HQ909306	CW3-12	β-Proteobacteria	Fig. 6
55	HQ909307	CW3-16	β-Proteobacteria	Fig. 6
56	HQ909309	CW3-25	β-Proteobacteria	Fig. 6
57	HQ909326	CW3-145	Acidobacteria	Fig. 5
58	HQ909316	CW3-130	α-Proteobacteria	Fig. 6
59	HQ909319	CW3-144	Firmicutes	Fig. 7
60	HQ909317	CW3-133	α-Proteobacteria	Fig. 6
61	HQ909318	CW3-136	β-Proteobacteria	Fig. 6
62	HQ909327	CW3-20	α-Proteobacteria	Fig. 6
63	HQ909330	CW3-167	β-Proteobacteria	Fig. 6
64	HQ909311	CW3-138	Firmicutes	Fig. 7
65	HQ909308	CW3-88	β-Proteobacteria	Fig. 6
66	HQ909312	CW3-33	α-Proteobacteria	Fig. 6
67	HQ909314	CW3-52	β-Proteobacteria	Fig. 6
68	HQ909320	CW3-147	α-Proteobacteria	Fig. 6
69	HQ909322	CW3-161	β-Proteobacteria	Fig. 6
70	HQ909332	CW3-142	Acidobacterium	Fig. 5
71	HQ909337	CW3-45	γ-Proteobacteria	Fig. 6
72	HQ909335	CW3-11	α-Proteobacteria	Fig. 6

#### Acidobacteria

Similarity search in NCBI and RDP databases indicated that all the *Acidobacterial* sequences had strong identity with uncultured members of this phylum. Particularly, sequences from non-contaminated samples showed a strong similarity with uncultured *Acidobacteria* from undisturbed mixed grass prairie and rice field soils. In contrast, *Acidobacteria* sequences retrieved from contaminated samples showed high similarity with uncultured clones mostly obtained from hydrocarbon-contaminated soil and sediment, soil adjacent to silage storage bunker and Altamira cave, etc. In order to determine the distribution of *Acidobacteria* sequences retrieved from ten clone libraries into different subgroups, a Neighbor-joining tree was constructed using representative *Acidobacteria* sequences of different subgroups along with our sequences (Fig. 5). Representative sequences for various subgroups were obtained from previously published works of Sait et al. 2006 (subgroup 1) and Hugenholtz et al. 1998 (subgroup 2, 3, 4, 5, 6, 7 and 8). Hugenholtz et al. (1998) mentioned that *Acidobacteria* subdivisions 1, 3, 4, and 6 are well represented by environmental clone sequences with only five cultured representatives. In this regard, our analysis indicated that members of subgroup 1, 3, 4 and 5 were present within all eight libraries. Members that belong to other subgroups (2, 6, 7 and 8) were not detected in the present libraries. Phylogenetic tree further revealed that the most dominant *Acidobacteria* clones from libraries B209, B210, T219, T221 and TD222 were present within subgroup 1. Within this subgroup, seven sequences representing the major ribotypes from the above libraries were included. Sequence of the clone B210-44 although shared identity with an uncultured bacterium, showed a close phylogenetic lineage with culturable representative *Acidobacterium capsulatumi* with 90% bootstrap value. The next cluster that accommodated maximum numbers of ribotypes showed their affiliation to subgroup 4. In this subgroup, four *Acidobacteria* clones from the contaminated site CW1, and three from non-contaminated sites (T112, B209 and B210) were included. Subgroup 5 was composed of four dominant *Acidobacteria* clones; two retrieved from contaminated library CW3 and other two from non-contaminated library T112. Subgroup 3 was represented by members from the library T222. Abundance of *Acidobacteria* in uranium-contaminated samples was reported by Barns et al. 2007. The ubiquity and abundance of *Acidobacteria* in soils and their ability to withstand polluted and extreme environments suggest that they serve functions important in the environment and are potentially quite varied (Ward et al. 2009).

#### Proteobacteria

Predominance of phylum *Proteobacteria* was observed mainly in contaminated samples, although their presence was detected within non-contaminated samples as well. Among the *Proteobacteria*, members of the class *β*-*Proteobacteria* was found to represent the abundant ribotypes in contaminated as well as non-contaminated samples. As evident from Fig. 5, within the contaminated samples nine ribotypes of CW3 library (42% coverage), five ribotypes (29% coverage) from CW2 library and two ribotypes (11% coverage) of library CW1 were affiliated to *β*-*Proteobacteria*. Several major ribotypes from non-contaminated samples (B18, B211 and B214) were belonging to *β*-*Proteobacteria*. As evident from the tree topology, three clones (CW3-63, CW3-161 and CW3-52) from CW3 formed a separate monophyletic clade showing their relatedness with thiosulfate oxidizing autotrophic bacterium *Thiobacillus thioparus* (ATCC 8158). In another clade clone B218-35 (covering 9% of the library) showed affiliation with N_2_-fixing bacterium *Herbaspirillum* sp*.* MS-F-86 (FJ460080) isolated from Ninghai harbor, China. In the same clade clone, CW3-136 and CW3-166 of library CW3 showed strong phylogenetic affiliation and lineage (100% bootstrap support) with nickel-resistant *Massilia timonae* (AY512824). Clone CW3-12 showed a strong affiliation with uncultured *β*-*Proteobacteria* (EF492892) isolated from soil around iron-manganese nodule from China. Other clones in the same library formed a single clade showing their affiliation with hydrocarbon degrader *Propionivibrio limicola* (AJ307983). Clone CW2-44 and CW3-25 showed affiliation with perchlorate-reducing bacterium *Dechloromonas* sp*.* LT-1 (AY124797) and denitrifying *Azonexus fungiphilus* (AF011350), respectively. Clone B214-3, the most abundant ribotype (covering 22%) of the library B218 formed a monophyletic node showing close relatedness with ‘nanometric’ bacterium *Ramlibacter tataouinensis* (AF144383) isolated from meteorite-impacted subdesert soil in Tunisia, France. The other node was comprised six clones. Clones B218-167 and B211-10 of this cluster showed a strong affiliation with uncultured *β*-*Proteobacteria* (AY921995). Clone CW3-167 showed affiliation with cholesterol-oxidizing, denitrifying bacterium *Sterolibactrium denitrificans* (AJ306683). In another clade, two clones CW1-8 and CW1-78 together covering 11% of the CW1 library showed affiliation with *Methylibium* sp*.* (EU130974) isolated from granular activated carbon filters. Along with clones CW2-75 and CW2-162 having relatedness with *Burkholderiales* bacterium YT0099, the later clones showed a strong lineage with members of *Burkholderiaceae* capable of redox transformation of metals to satisfy metabolic requirements. Sequence of clone CW3-88 formed a separate clade showing its distinctness from other members but strong phylogenetic lineage (99% bootstrap value) with uncultured *Alcaligenaceae* (AM936094) isolated from hydrocarbon contaminated soil. Members of the class *γ*-*Proteobacteria* were relatively more abundant in contaminated samples. Sequence of clone B218-15 was highly related to an uncultured member of *γ*-*Proteobacteria*. Two clones (CW2-3 and CW2-128 covering 21% of CW2 library), showed a strong phylogenetic affiliation with arsenic-resistant soil bacterium (AF308875). Sequence of clone B210-14 marked its affiliation with uncultured *Xanthomonadace* bacterium, while two clones CW1-36 and CW3-45 showed their lineage to polychlorinated dioxin dechlorinating bacterium. Members of the class *α*-*Proteobacteria* were represented by ribotypes from contaminated samples CW2 and CW3, covering 25 or 27% in respective libraries. Within this class, sequences of clones CW2-52 and CW2-77 showed a strong relatedness with N_2_-fixing bacteria *Azospirillum amazonense.* Sequences of clones CW3-33 and CW1-36 showed phylogenetic affiliation with uncultured bacterium isolated from floor dust. Together with sequence of CW2-29, both these two clades were found to be related sharing the same node, thus indicating a common phylogenetic origin. The remaining clade within this class was composed of five clones. Clone CW3-130 and CW3-147 (covering 7% of the CW3 library) showed affiliation with mixed grass bacterium where as clone CW2-100 showed affiliation with *Microvirga* sp. (EU529844) isolated from soil of cucumber farming greenhouse. Sequence of clone CW2-140 showed a strong affiliation with *Parvibaculum lavamentivorans* (AY387398) bacterium capable of alkylbenzenesulfonate surfactant (LAS) biodegradation. Sequences of three other clones CW3-133, CW3-20 and CW3-11, although strongly related to each other showed phylogenetic affiliation with uncultured bacterium isolated from a poplar tree microcosm. Class *δ*-*Proteobacteria* was represented by two clones (B218-10 and B211-9). Sequence of clone B218-10 was the most abundant ribotype in library B218 that showed affiliation with *Desulfovibrio alkalitolerans* (AY649785) isolated from a heating water of Denmark. Dominancy of *Proteobacteria* in contaminated sites is well reported (Rastogi et al. 2010; Ellis et al. 2003). Lineages belonging to *Proteobacteria* are well known to survive in oligotrophic environments, capable of metal reduction and resistance and play very important roles in uranium immobilization in contaminated sediments or groundwater.

#### Bacteroidetes, Firmicutes, Actinobacteria, Gemmatimonadetes and Cyanobacteria

Members of these phyla were more abundant in non-contaminated samples (Fig. 6). Phylum *Bacteroidetes* was represented by five ribotypes. Sequence of clone B209-3 showed a strong phylogenetic lineage (100% bootstrap value) with *Flavobacterium* sp. (EU194891) while clone (B211-39) showed a strong affiliation with uncultured bacterium isolated from gold mine tailings of Nederland. Clone CW2-129 showed affiliation with *Sediminibacterium* sp. (FM179320) where as clone CW1-57 showed affiliation with uncultured bacterium isolated from a waste-gas biofilter. Presence of the phylum *Bacteroidetes* in heavy metal and uranium-contaminated soils and sediments were reported previously by Ellis et al. 2003; Reardon et al. 2004; Brodie et al. 2006 and Akob et al. 2008.

Phylum *Cyanobacteria* was represented by eight clones. Among the four clones from non-contaminated sites, three from library T219 showed lineage with *Oscillatoria duplisecta* (AM398647) isolated from a thermal mud of Euganean thermal springs (Italy). Clone CW2-104 from sample CW2 also showed affiliation with *Oscillatoria acuminata* (AB039014). Other three clones (CW2-96, CW2-34 and CW2-35) from contaminated sample CW2, covering 11% of the clone library, and showed affiliation with uncultured *Chlorophyta* chloroplast 16S rRNA gene (AB374385). *Cyanobacteria* were the ubiquitous group present in most soil habitats and possessing high tolerance to environmental stress (Chong et al. 2010). Our results indicated that it may be present both in contaminated and non-contaminated samples. It also can act as a biosorbent of heavy metals in sewage water (El-Enany and Issa 2000). Phylum *Firmicutes* was represented by four clones. Clone B211-27 showed a strong phylogenetic affiliation (100% bootstrap value) with *Firmicutes* bacterium isolated from Pere Marquette River sediment. Clone B219-8, CW3-144 and CW3-138 showed a strong phylogenetic affiliation (100% bootstrap value) with Manganese (II)-oxidizing *Bacillus* sp. Akob et al. **(**2008) showed the presence of *Firmicutes* in radionuclide-contaminated subsurface sediments during the early part of the ethanol microcosm incubation suggesting that these groups may have actively involved in nitrate reduction and subsequent uranium reduction both important for the immobilization of U(VI) in contaminated sediments. Merroun et al. (2005) showed that *Bacillus* sp., can be recovered from uranium mining waste pile and can make complexion with uranium by either whole cells or S-Layer sheets. Phylum *Chloroflexi* was mainly represented by ribotypes from sample B214. Sequences of clone B214-22 and B214-6 formed a distinct monophyletic clade along with *Anaerolinea thermolimosa* (AB109437) isolated from thermophilic granular sludge. Phylum *Actinobacteria*, *Nitrospira* and *Gemmatimonadetes* were all represented by single ribotype. In the phylum *Actinobacteria*, clone CW2-97 was found to be phylogenetically affiliated (with 100% bootstrap value) with *Cellulomonadaceae* str. KB8 isolated from paddy rice. Clone B209-18 showed a strong phylogenetic affiliation with nitrite oxidizing *Nitrospira moscoviensis*. In the phylum *Gemmatimonadetes*, clone CW1-14 showed affiliation with uncultured bacterium isolated from oil palm empty fruit bunches compost heap.
